# Is the SARS CoV-2 Omicron Variant Deadlier and More Transmissible Than Delta Variant?

**DOI:** 10.3390/ijerph19084586

**Published:** 2022-04-11

**Authors:** Bao V. Duong, Puchanee Larpruenrudee, Tianxin Fang, Sheikh I. Hossain, Suvash C. Saha, Yuantong Gu, Mohammad S. Islam

**Affiliations:** 1 School of Mechanical and Mechatronic Engineering, University of Technology Sydney, Ultimo, NSW 2007, Australia; baovuong.duong-1@alumni.uts.edu.au (B.V.D.); puchanee.larpruenrudee@uts.edu.au (P.L.); tianxin.fang-1@student.uts.edu.au (T.F.); suvash.saha@uts.edu.au (S.C.S.); 2 School of Life Sciences, University of Technology Sydney, Ultimo, NSW 2007, Australia; sheikhimamul.hossain@uts.edu.au; 3 School of Mechanical, Medical and Process Engineering, Faculty of Engineering, Queensland University of Technology, Brisbane, QLD 4000, Australia; yuantong.gu@qut.edu.au

**Keywords:** COVID-19, SARS-CoV-2, Delta variant, Omicron variant, variants of concern, transmissibility, severity and mortality

## Abstract

Genetic variants of severe acute respiratory syndrome coronavirus (SARS-CoV-2) have been globally surging and devastating many countries around the world. There are at least eleven reported variants dedicated with inevitably catastrophic consequences. In 2021, the most dominant Delta and Omicron variants were estimated to lead to more severity and deaths than other variants. Furthermore, these variants have some contagious characteristics involving high transmissibility, more severe illness, and an increased mortality rate. All outbreaks caused by the Delta variant have been rapidly skyrocketing in infection cases in communities despite tough restrictions in 2021. Apart from it, the United States, the United Kingdom and other high-rate vaccination rollout countries are still wrestling with this trend because the Delta variant can result in a significant number of breakthrough infections. However, the pandemic has changed since the latest SARS-CoV-2 variant in late 2021 in South Africa, Omicron. The preliminary data suggest that the Omicron variant possesses 100-fold greater than the Delta variant in transmissibility. Therefore, this paper aims to review these characteristics based on the available meta-data and information from the first emergence to recent days. Australia and the five most affected countries, including the United States, India, Brazil, France, as well as the United Kingdom, are selected in order to review the transmissibility, severity and fatality due to Delta and Omicron variants. Finally, the vaccination programs for each country are also reviewed as the main factor in prevention.

## 1. Introduction

There is no doubt that the entire world’s population has been suffering from coronavirus disease (COVID-19) pandemic since the first infection of a new coronavirus, recently known as severe acute respiratory syndrome coronavirus 2 (SARS-CoV-2), which was recorded in Wuhan, China in December 2019 [1,2,3]. More importantly, it has caused unimaginable damages to societies and economies worldwide due to its increased transmissibility and high mortality rate [4,5,6,7,8]. In general, air pollutants with micro-particle (PM10, PM2.5, and other pollutants in the atmosphere) can go deep into the alveolar region of the human lung and lead to severe respiratory diseases. However, the amount of this particle deposition is based on the lung sizes (human age), daily physical activities (causing different airflow rates), and micro-particle sizes [9,10,11,12,13,14,15,16]. For SARS-CoV-2, it is classified as a nano-particle at 120 nm and can be up to 1000 nm [17,18,19]. Several researchers proved that toxic nano-particles lead to a greater negative impact on the respiratory system compared to toxic micro-particles [20,21,22,23].

According to the latest updated data from the World Health Organization (WHO) [24] on 26 March 2022, the world has recorded over 480 million COVID-19 cases, and it has been responsible for over 6 million deaths around the world. Unfortunately, since the pandemic, SARS-CoV-2 has been dramatically evolving in a wide range of variants dedicated to devastating characteristics [25,26,27,28]. Apart from this, the term “variant” refers to a group of viruses that have mutated from the original lineages with additional changes to the characteristic spike protein [29]. According to The United States Department of Health and Human Services (HHS) and SARS-CoV-2 Interagency Group (SIG) [30], there are three main types of variant classifications, including Variant of Interest (VOI), Variant of Concern (VOC) and Variant of High Consequence (VOHC). In terms of VOCs, many countries have been struggling with the emergence and spread of ongoing variants, including in Southeast Asia, Middle East, Europe and North America. A group of experts at the World Health Organization (WHO) decided to name these variants using the Greek alphabet, such as Alpha, Beta, Gamma and Delta, which is easier for non-scientific people to discuss [31]. According to WHO [32], some of the VOCs are listed in Table 1 below:

In terms of the Delta variant of SARS-CoV-2, it has caused the latest wave of infection resulting in socio-economic setbacks across many countries around the world [33]. In December 2020, the first case of the Delta infection was detected in India [34,35,36,37,38]. Since then, the Delta variant has been drastically spreading across many unvaccinated countries with tremendous numbers of cases, increasing hospitalizations and mortality rates [34,39,40,41]. However, Del Rio et al. [34] also presented that the emergence of the Delta variant in the UK and US have created a massive wave of new infections despite their high vaccination rate [34]. Noticeably, the most affected areas are highly located at unvaccinated or delayed second-dose communities. Generally speaking, some main factors should be taken into account to discuss the characteristics of the Delta variant. First, its transmissibility is highly infectious, which is more than double compared to previous lineages. Second, Sheikh et al. [42] and Fisman [43] claim that in unvaccinated patients, the infections of the Delta variant potentially cause more severe sickness than previous variants [42,43]. Additionally, these patients are highly likely to be monitored under the intensive care unit (ICU) than from the Alpha and original strains. Moreover, Riemersma et al. [44] and Nasreen et al. [45] analysed the impacts of the Delta variant on fully vaccinated people, and they agree that although there are breakthrough infections among the vaccinated people, the infections tend to reduce faster than unvaccinated people. 

Regarding the outbreaks in Australia, Australia has undoubtedly succeeded in preventing the initial wave of infection (March–June 2020) and second wave (June–September 2020) in the south-eastern state of Victoria (VIC) by their effective actions, including quick and immediate management in contact tracing and local lockdowns, travel restrictions, and social distancing [46,47,48]. Despite the trigger of social distancing restrictions and business shutdowns involving retailers and construction work, the number of confirmed cases has significantly risen to 100 and 439, within 10 days and 23 days, respectively, since the first case was detected [49]. It is evident that the Delta variant is more transmissible than previous variants, regardless of if it can result in more severity and mortality. First, many studies suggest that unvaccinated people are the most vulnerable because they tend to be sicker quickly and more severely during the infection. An instance illustrating this point is that the UK study asserts that Delta cases have double the risk of hospitalization than Alpha cases among over 40,000 SARS-CoV-2 cases [40]. Furthermore, a study in Canada also claims that infected people by the Delta variant escalate the probability of hospitalization and fatality by twofold [47]. Consequently, transmissibility and mortality are the most concerning characteristics of the Delta variant.

Conversely, the detection of a new and greatly mutated SAR-CoV-2 variant in South Africa, Omicron, has been causing global grave concern. First, the Omicron variant is designated as a variant of concern, B.1.1529, by The World Health Organization (WHO) [50], and it was first reported in Botswana on 11 November 2021. Since the initial detection, the Omicron variant has spread in over 20 countries across the globe within a week, including in Hong Kong, the UK, the US and Australia. Thus, Omicron is globally recognized as a high-risk variant. Omicron has currently become the most predominant variant detected in SARS-CoV-2 cases in various South African countries, such as Botswana and Zimbabwe, and in Israel and Hong Kong. According to Rao and Singh [51], the Omicron variant is 100-fold greater, as compared to the Delta variant, in the rate of infection. In detail, a few recent studies were conducted to analyse the mutational profile of the Omicron variant [52,53]. Therefore, Venkatakrishnan et al. [52] claim that after the comparison between Omicron and previous variants of concern (Delta, Alpha, Gamma, Delta), the Omicron variant possesses up to 37 mutations in its spike protein involving 26 unique mutations which make Omicron a “step function” of the SARS-CoV-2 strains’ evolution. The summary of the mutational profile of the spike mutations in the SARS-CoV-2 variants of concern can be found in Table 2.

Furthermore, there is a well-established fact that the Omicron variant has higher transmissibility and infectivity than the Delta variant [54,55,56]. However, there is no available evidence that the Omicron variant can cause more severe sickness or that it can reduce vaccine effectiveness. In brief, due to the deadly continuing spread of Omicron and Delta VOCs worldwide, many countries, including the United States (US), Canada, and the United Kingdom (UK) have been conducting several studies to investigate national and global trends of these two VOCs infected waves. However, there are limited studies conducted to analyse not only the highlighted characteristics but also the differences between Omicron and Delta VOCs, in terms of transmissibility and severity.

Therefore, this review paper aims to summarize the differences between Delta and Omicron variants by focusing on the impact of Omicron and Delta variants on vaccine effectiveness, transmissibility, severity, mortality. To provide more details, Australia and the most affected countries including Brazil, France, India, the US and the UK will be selected for this review. In addition, this paper also summarizes the current preventions that have been used to deal with SARS-CoV-2 variants.

## 2. SARS-CoV-2, Delta Variant and Omicron Variant

### 2.1. Chemical Composition

**Figure 1:**
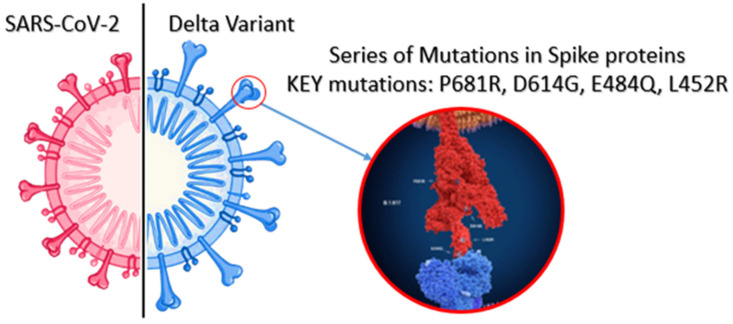
Comparison structure of SARS-CoV-2 and Delta variant.

**Figure 2:**
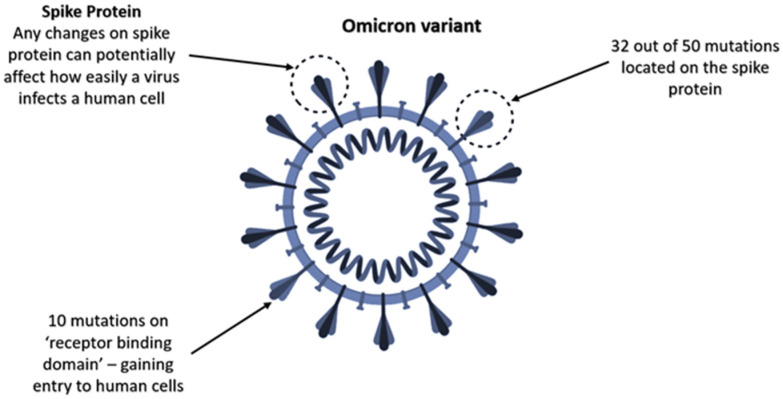
The new COVID-19 variant: Omicron (B.1.1.529).

Many countries have been experiencing the global emergence of SARS-CoV-2 (VOCs), predominantly Delta variant, since December 2020. Therefore, several studies have been conducted to analyse the mutated spike variants of SARS-CoV-2 [25,26,27,28]. According to the Centers for Disease Control and Prevention [31], SARS-CoV-2 viruses have constantly been changing over time, and this process is called mutation [57]. Moreover, it normally results in the emergence of new variants. In simple terms, this process can be described as the growth of trees and branches. When the tree grows up, branches also grow up together. Additionally, while some of the branches grow and die off, others may be strong enough to overcome blocks and spread quickly [57]. Similarly, COVID-19 mutates into emerging new variants, and some of the variants pose new mutated characteristics, such as easier binding to human cells, rapid reproduction and resisting antibodies or treatment. When it comes to the Delta variant, it has been determined that it has some significant mutations in the spike protein of the virus structure compared with the original lineages [58,59,60,61,62,63]. In Figure 1, there are some main mutations in the spike proteins of the Delta variant, including E484Q, L452R, P681R and D614G [59]. More importantly, these mutations play a crucial role in making the Delta variant more transmissible. It can be explained by the fact that these mutations can unlock the human cells and even evade natural human immunity. However, due to the recent emergence of the Omicron variant in late 2021, there are limited studies investigating the mutation profile of this variant. Specifically, Venkatakrishnan et al. [52] claim that the Omicron variant possesses up to 26 unique mutations and seven overlapped mutations between the Omicron and Alpha variants after comparing 37 mutations in the Omicron variant to other variants (Figure 2). Furthermore, although variants with a higher number of mutations are not potentially more dangerous, the Omicron variant with 32 out of 50 mutations located on the spike protein is more likely able to affect how easily it infects a human cell. 

### 2.2. Delta Variant and Omicron Variant Outbreaks

**Figure 3:**
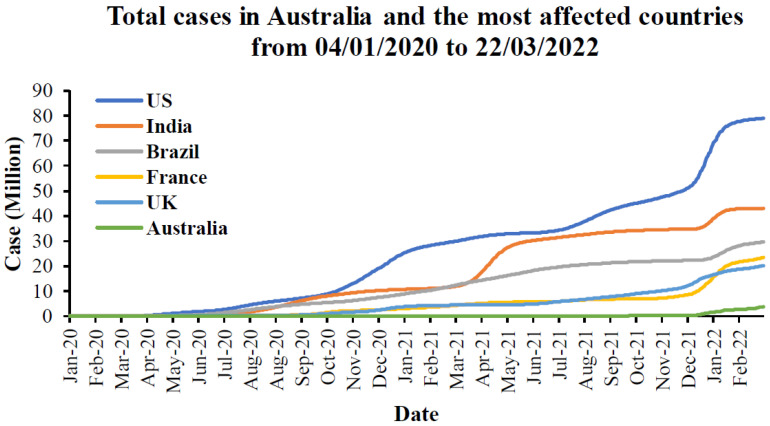
Total cases in Australia and the most affected countries from 4 January 2020 to 22 March 2022 [64].

**Figure 4:**
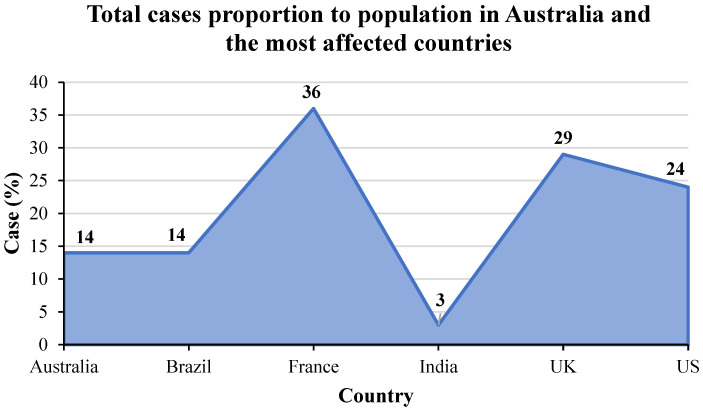
Total cases in proportion to population in Australia and the most affected countries from the first emergence to March 2022.

The total cases in Australia and the most affected countries from the first emergence to March 2022 are provided in Figure 3 based on the latest updated data from the WHO [64]. From this Figure, it can be seen that the US is the most affected country by SARS-CoV-2, followed by India, Brazil, France, and the UK, in order. Australia is the least affected country compared to the total cases since the first to recent days. The cumulative cases in the US are approximately 80 million, while there are around 30 to 20 million cases in other countries except for Australia. For Australia, there are around 4 million cases that have been caused by this pandemic. When focusing on the proportion of the total cases to the population in these countries in Figure 4, France is the most affected country followed by the UK and the US, while Brazil and Australia have the proportion of the same cases to population. India has the lowest number of cases compared to other countries.

Focusing on the Delta variant, it is evident that the infected wave of the coronavirus epidemic is predominantly attributed to the emergence of the Delta variant of SARS-CoV-2 across 98 countries, such as India, the UK, the US and Southeast Asian countries in 2021. The main reason for the rapid spread of the Delta variant is its transmissibility, which is estimated at approximately more than 60% compared with the Alpha variant. Meanwhile, since the first discovery of the Omicron variant in South Africa in November 2021, Omicron has been circulating in over 63 countries, and has become dominant in Australia, the United Kingdom and the United States. Consequently, to control the current spread, it is necessary to understand the transmissibility of the Delta and Omicron variants by analysing recent outbreaks throughout the world [65].

**Figure 5:**
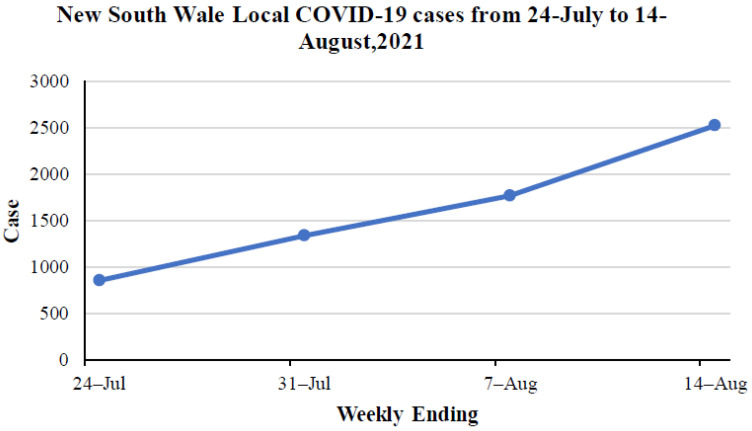
New South Wales local COVID-19 cases from 7 to 14 August 2021 (accessed 14 August 2021) [66].

**Figure 6:**
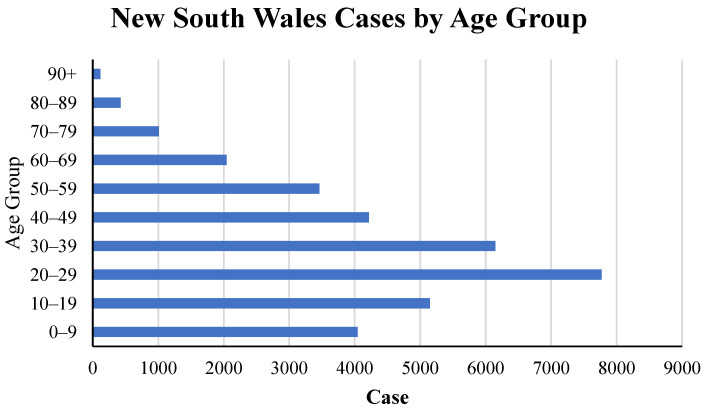
New South Wales cases by different age groups (accessed 8 September 2021) [66].

When it comes to Australia, Sydney has been suffering from a wave of Delta variant infections since July 2021. Australia’s latest outbreak was caused by the Delta variant, with the first case detected in Sydney on June 16. The outbreak then quickly spread to other states. According to the recorded data from NSW Health [66], which is the official NSW government website, the specific period was selected and is provided in Figure 5. This figure illustrates that 2521 locally acquired cases have been reported since 14 August 2021. As can be seen from that time, the number of confirmed cases has suddenly increased. In terms of time, the further in time, the more obvious the upward trend is. During the first week (from 24 to 31 July), the number of cases increased to 484, while in the second week (from 31 July to 7 August), the case numbers were 429, and in the third week (from 7 to 14 August), it skyrocketed to 754 cases. From this period, it is obvious to see that the transmission rate is fast. Conversely, in Sydney, during the current Delta infection, the number of cases in young people, with ages involving 10–19, 20–29 and 30–39, account for the highest infections, at approximately 5000, 8000 and 6000, respectively (Figure 6). Similarly, the 0–9 age group also has a great number of cases, which is around 4000 (Figure 6). In simple terms, the higher transmissibility of the Delta variant is demonstrated by the dramatic infections of the younger ages, even in the 0–9 age group, which is certainly believed to be immunized to the original lineage. 

**Figure 7:**
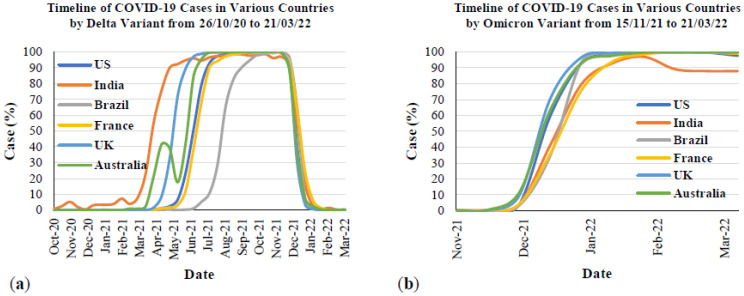
The timeline of SAR-CoV-2 cases in Australia and the most affected countries from 26 October 2020 to 21 March 2022: (**a**) Delta variant; (**b**) Omicron variant.

Figure 7 presents the timeline of the Delta and Omicron outbreaks in Australia and the five most affected countries from the first emergence to March 2022. This figure was created based on the timeline for each variant from the beginning until obtaining 100% of the total cases in each country. Figure 7a is the timeline of the Delta variant, and Figure 7b presents the timeline of the Omicron variant. The data from this figure were taken and recreated from Our World in Data [67]. This organization is a collaborative effort between researchers at the University of Oxford and the non-profit organization, Global Change Data Lab. From Figure 7a, the Delta variant was first detected in India in October 2020 and rapidly spread by May 2021. After that, the COVID-19 cases caused by this variant were stable and rapidly decreased by December 2021. Other countries were also affected by the Delta variant in different periods. However, the trends from all countries were similar to India, in that they rapidly increased and remained stable until December 2021. Regarding Figure 7b, COVID-19 cases by the Omicron variant rapidly increased within a month starting from December 2021, which is the end of the COVID-19 cases caused by the Delta variant. More information will be provided in the transmission section. For more analysis, if considering the timeline of COVID-19 cases caused by the Delta variant in all six countries (Figure 7a), it can be seen that the total number of cases (Figure 3) significantly increased, with a 100% case rate of this variant. However, if compared to the timeline of COVID-19 cases by the Omicron variant (Figure 7b), between December 2021 and January 2022, the total cases (Figure 3) rapidly increased in this period, which is the same time as when this variant reached 100% of total cases.

### 2.3. Transmission

#### 2.3.1. Respiratory Droplet Transmission

**Figure 8:**
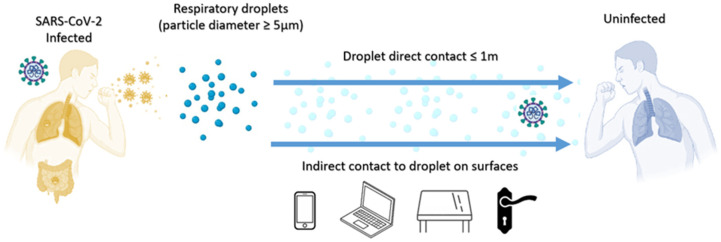
Delta variant droplet transmission.

Reports show that the virus can survive on the surface of respiratory droplets, secretions, and contaminated bodies for a while. When a patient coughs or expects sputum, the droplets usually contain many viruses, which can stay on the surface for a while, such as elevator buttons, stair handrails, or the surface of express parcels (Figure 8). When healthy people touch these contaminated objects, if they do not wash their hands properly and touch their eyes, nose, and mouth, they will be infected. For example, someone who touched the surface of an object touched by a confirmed patient with his hand would then be infected with the virus when touching his mouth, eyes, or nose. When people sneeze, cough or even talk, the virus they carry can infect people who are in close contact with them. It is easier for air to flow in outdoor spaces, and it is difficult for outdoor spaces to form air circulation. These minimize the theoretical risk of aerosol transmission through smaller respiratory droplets. Outdoor spaces usually allow for more physical distances, thus reducing the risk of transmission of the virus through large respiratory droplets [68]. The main way humans are infected with the Delta variant is through contact with respiratory fluids that carry infectious viruses. The risk of spreading the SARS-CoV-2 Delta variant in an outdoor environment is low [69].

#### 2.3.2. Transmission by Exposure to Contaminants

The Delta variant can be spread through objects. In patients infected with the Delta variant, the virus appears earlier in the body, the amount of virus is higher, and the amount of virus is higher than the original new coronavirus. The virus remains positive in the patient’s body for a longer time than the original virus strain [70]. Therefore, all these factors can greatly increase the speed of the Delta virus spreading through surfaces.

#### 2.3.3. Airborne or Aerosol Transmission

**Figure 9:**
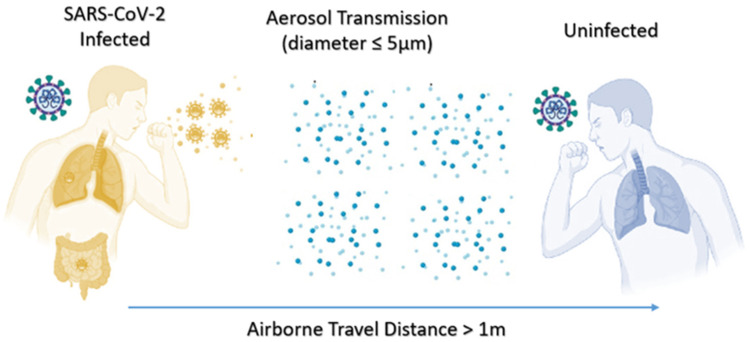
Delta variant aerosol transmission.

Airborne transmission is also called “aerosol transmission”. People should pay special attention, especially indoors, in closed and unventilated narrow spaces [71]. If an infected person coughs or sneezes, small respiratory secretion particles will form (Figure 9). These particles can be suspended in the air. Inhalation can cause infection. Although the transmission of aerosols in the community environment is controversial, new data show that indoor circulating air can transmit SARS-CoV-2, for example: an incident of spreading at an indoor choir practice in Washington State, USA [72], a meat processing plant in the US [73], and a nursing home in the Netherlands [74]. In low-ventilated areas, the atomized droplets can stay suspended longer before being inhaled or falling onto a surface, which may lead to the spread of contaminants. In a closed environment, low humidity, air conditioning, and low ultraviolet light may all help the virus particles survive longer [68]. Especially for the ventilation of small indoor spaces, if someone coughs or sneezes, small particles of respiratory secretions can be formed and suspended in the air. Conversely, it may cause infection or even remove the virus and leave the space. The virus in the air still exists for a certain time, causing other people to be infected. The proven effective anti-SARS-CoV-2-Delta transmission strategy also includes diluting the air through maximize ventilation and filtration [69].

#### 2.3.4. Animal to Human

The spread of the virus depends on the host, and the infection of the virus to the host is selective. Viruses are classified into plant viruses, animal viruses and bacterial viruses according to the organisms they live in. Viruses that live exclusively in plant cells are called plant viruses, such as tobacco mosaic virus; viruses that live in animal and human cells are called animal viruses, such as influenza viruses and coronaviruses; viruses that specialize in bacteria are called bacteria viruses (also called bacteriophages), such as caliphates. In other words, a virus cannot infect every species. It depends on whether the virus can complete one generation in that species to produce a new generation of viruses. The virus itself has only a long string of nucleic acids and a protein shell. To complete the replication, transcription and translation of its genetic information, it must be carried out in the host cell, which requires the host cell to provide the material and energy it needs. Otherwise, the virus cannot complete replication and will not be able to infect.

In order to infect the host, the virus first needs to be able to bind to the host cell’s receptors such that it can use the host cell’s material and energy to synthesize its material. However, this is not always easy. Generally speaking, the closer the related species are, the more likely they are to combine, while the further the species are, the less likely it is to be infected. This is why plant viruses generally cannot infect animals.

#### 2.3.5. Human Behaviours—Sneezing without Wearing a Mask

Because the Delta virus load is high and the spread speed is fast, and the Delta virus can be spread through pollutants and air, droplets from sneezing, coughing and even talking may carry the virus and infect people who are in close contact with the virus [75]. Thus, when people do not wear masks and are in the same small, confined space as the confirmed patient, they are susceptible to transmission. The proven effective anti-SARS-CoV-2 Delta transmission strategy always includes the correct use of masks [68]. The CDC recommends that the community uses multilayer cloth masks or non-medical disposable masks. When it is not possible to maintain a physical distance of ≥6 feet, it is vital to use masks in indoor spaces and outdoors. In the family, masks should be used when family members are infected or have recent potential SARS-CoV-2 -Delta exposure (for example, known close contact or potential exposure related to occupations, crowded public places, travel, or non-family members) [70].

### 2.4. The Difference in Transmissibility between the Delta Variant and Omicron Variant

Alpha (B.1.1.7): the first variant of attention described in the UK in late December 2020. Beta (B.1.351): first reported in South Africa in December 2020. Gamma (P.1): first reported in Brazil in early January 2021. Delta (B.1.617.2): first reported in India in December 2020. Among these variants, symptoms of the Delta variant in infected patients may be slightly different. For example, headache, fever, sore throat and runny nose are common, but cough and loss of smell are not. Hearing loss is one of the new symptoms noticed among Delta variant patients [76]. 

**Figure 10:**
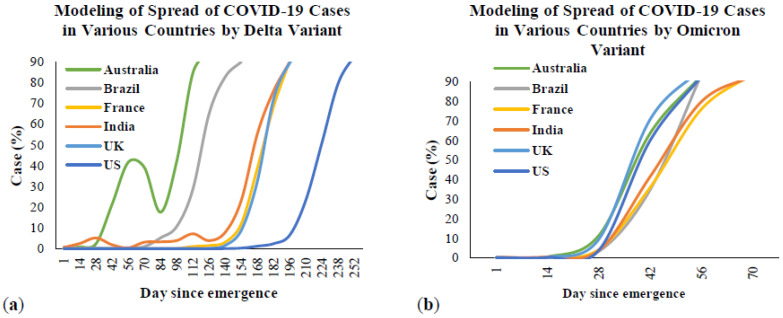
Comparison between Delta and Omicron outbreaks in the most affected countries and Australia: (**a**) cases by Delta variant; (**b**) cases by Omicron variant.

**Figure 11:**
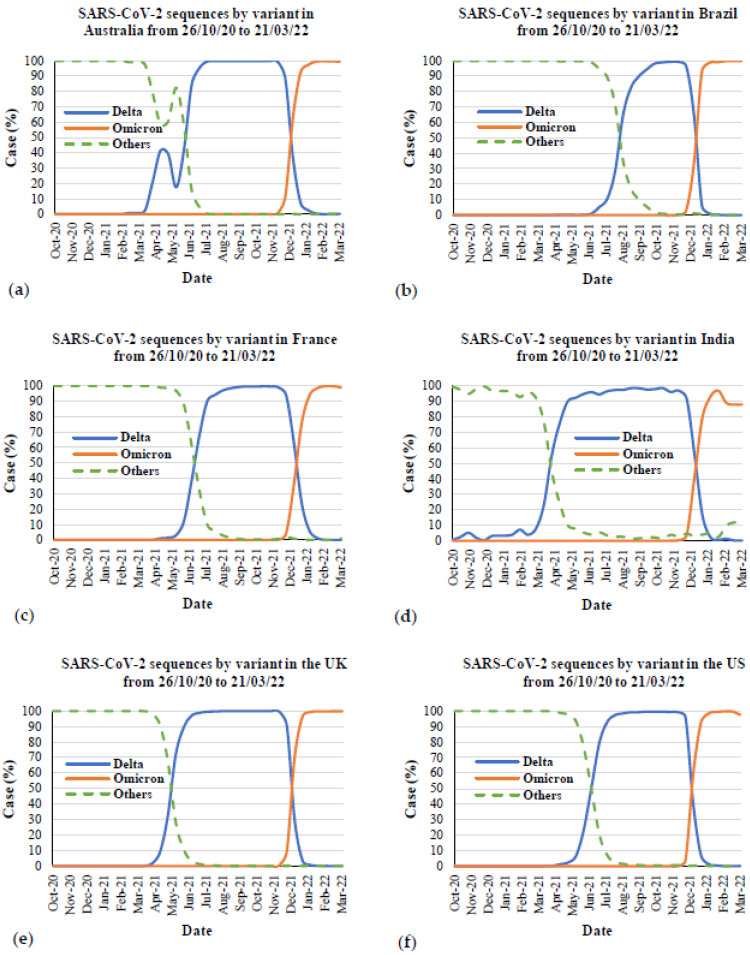
SARS-CoV-2 sequences by variant in Australia and the most affected countries from 26 October 2020 to 21 March 2022: (**a**) Australia, (**b**) Brazil, (**c**) France, (**d**) India, (**e**) the UK, and (**f**) the US.

Figure 10 presents the comparison between the Delta and Omicron variants in the most affected countries and Australia. Figure 11 presents the SARS-CoV-2 sequences by the Delta, Omicron and other variants in Australia and the five most affected countries from the first emergence in October to March 2022. The data for all countries were also taken from Our World in Data [67]. The first emergence case from the Delta variant was in October 2020 in India and November 2021 for the Omicron variant in South Africa. From this Figure, it is evident that the Delta and Omicron variants are the main causes of COVID-19 since the first emergence of these two variants. From the COVID-19 cases due to the Delta variant, it took over three months in all countries to reach 100% of cases caused by this variant since first emergence. After that, this variant remained stable at 100% of COVID-19 cases from all countries and rapidly decreased since November 2021. In contrast, COVID-19 cases due to the Omicron variant rapidly increased between December 2021 and January 2022. Focusing on the peak of the Delta outbreak with 80% to 100% cases in total, the peak outbreak in Australia (Figure 11a) was between June and December 2021, whereas in Brazil (Figure 11b), it was between September and December 2021. The peak outbreaks in France (Figure 11c), India (Figure 11d), and the US (Figure 11f) were between July and December 2021. For the peak outbreak in the UK (Figure 11e), it was from May to December 2021. From the meta-data, it can be seen that the beginning time of the peak outbreak is different in Australia, Brazil, and the UK, whereas the beginning of the peak outbreak in other countries is the same month of July 2021. This could be because of the different first emergences in each country. However, the end of the peak outbreak from Delta was found to be during the same month in December 2021, which was the same time as the rapid increase of cases by the Omicron variant. Focusing on the cases by the Omicron variant after reaching 100% cases in total, the trend of 100% cases was likely stable in all countries except for India. There were around 88% cases caused by the Omicron variant from February to March 2022, while fewer cases were caused by other variants (Figure 11d).

### 2.5. Severity and Mortality

**Figure 12:**
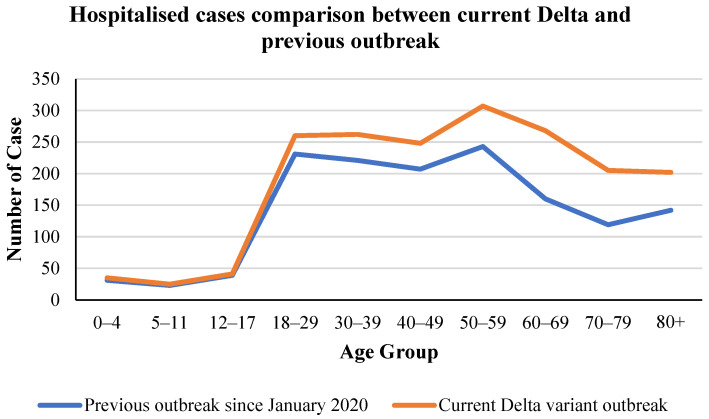
Hospitalized cases comparison by age group between the current Delta and previous outbreaks in Australia [66].

It is demonstrated in the previous section that the Delta variant was the most transmissible and has recently become the most concerning strain of COVID-19 in many countries around the world [77,78,79,80,81]. However, another question needed to be clarified: how severe and fatal is the Delta variant among infected patients. Several studies have suggested that the Delta variant can cause more severe health impacts on infected people in relation to severity and fatality. First, a study in the US stated that the Delta variant is deadlier than other variants [65]. Higher viral loads mean that different people can be infected in the same space, all in close contact, making it harder to control. Faster means the virus has a shorter disease incubation period, a shorter transmission time, and a faster transmission rate. The incubation period decreased from 5.9 days to 3.2 days on average. The interval between infection and infecting another person was even shorter, about two days on average. In addition, the study also showed that approximately 80% of COVID-19 Delta deaths occurred in patients over 65 years old [82]. For COVID-19 patients of any age, having underlying health conditions and underlying diseases also increased the risk of serious illness or death [70]. Apart from this, a British survey asserted that the death rate seemed to increase exponentially with age; men, obesity, socio-economic poverty, and many comorbidities are also associated with higher risks [83]. Furthermore, Sheikh et al. [42] conducted a cohort study in Scotland to compare the hospitalization rate between Alpha- and Delta-infected people. They claim that the Delta variant was 85% greater than the Alpha variant in hospitalization rate [42]. This is supported by Figure 12, showing the comparison between hospitalized cases of the previous outbreak since January 2020 and the current Delta variant outbreak within different age groups in Australia. In Figure 12, there is no difference between the age groups of 0–4, 5–11 and 12–17 hospitalized cases. However, for the groups from 18 to 80+ ages, the number of hospitalized cases in the Delta variant outbreak is higher than in the previous outbreak, at approximately 20%. 

**Figure 13:**
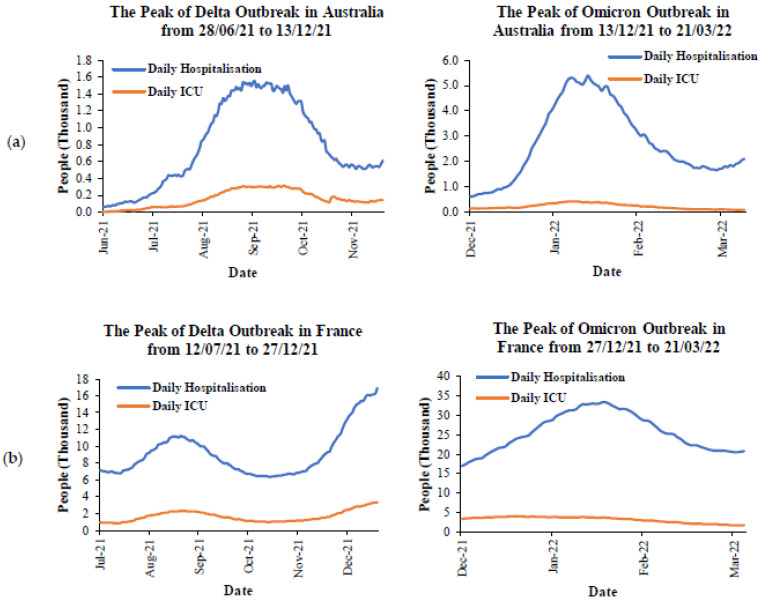

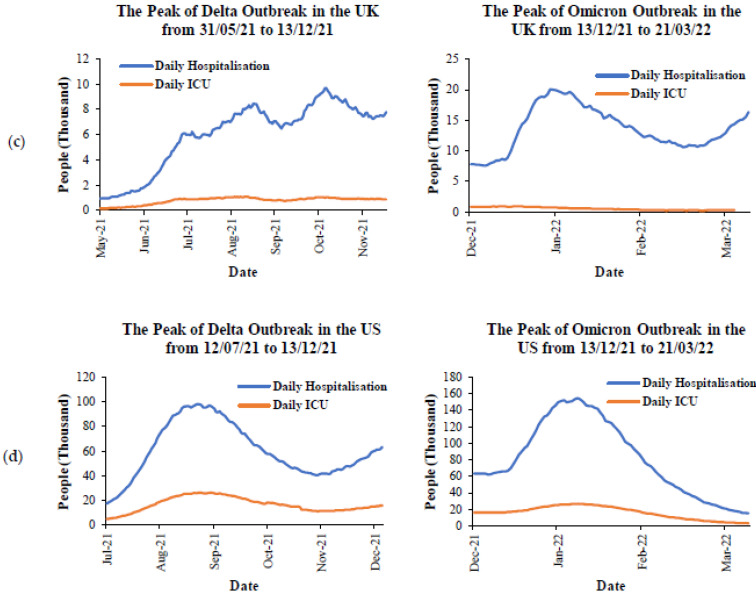
The comparison of severity during the peaks of Delta and Omicron outbreaks in: (**a**) Australia, (**b**) France, (**c**) the UK, and (**d**) the US.

To understand the impact of the Delta and Omicron variants on severity, the peak of the Delta and Omicron outbreaks in each country from Figure 11 are selected by focusing on the range between 80% to 100% cases in total for each variant. Figure 13 presents the daily cases of hospitalization and ICU during the peak of the Delta and Omicron outbreaks in Australia and the five most affected countries. The data for daily hospitalization and ICU cases were taken from the government websites in each country [66,83,84,85]. During the peak outbreak of the Delta variant in Australia (Figure 13a), there were less than 200 people minimum and around 1400 people maximum for daily hospitalization. For ICU cases, there were around 200 people maximum for daily ICU rates. However, when compared to the peak outbreak of the Omicron variant, there were around 5000 people maximum and less than 100 people minimum of daily hospitalizations. It can be seen that the maximum daily hospitalization during the peak of the Omicron outbreak was significantly higher than during the Delta outbreak at three instances. For the daily ICU cases, the maximum number of cases due to the Omicron outbreak was less than 500 cases, but it was still higher than the maximum number of cases due to the Delta outbreak around one time. In terms of France (Figure 13b), the maximum and minimum daily hospitalization rates during the peak outbreak due to the Omicron variant were found to be around one time higher than the cases by the Delta variant. For the daily ICU cases, the maximum number of cases during the Omicron outbreak was found to be higher than the cases during the peak Delta outbreak, with around 600 people, which is less than one time. Similarly, the daily hospitalization cases during the peak outbreak from the Omicron variant in the UK (Figure 13c) and the US (Figure 13d) were observed to be around one-time higher than the cases during the peak outbreak from the Delta variant. These were observed for both minimum and maximum daily cases. However, the maximum daily ICU cases during the peak outbreak from these variants were similar. Furthermore, the trend of daily ICU cases was found to be reduced in all countries. In contrast, the trend line of daily hospitalization cases was found to have fluctuated for all countries except for the US, which was reduced. To summarize, the Omicron variant significantly affected the daily cases of hospitalization, and it was around one to three times the daily cases from the Delta variant. However, the Omicron variant had less effect on the daily ICU cases. This was probably due to the total number of vaccinated people in each country. The discussion of this factor is provided in the Prevention section. 

**Figure 14:**
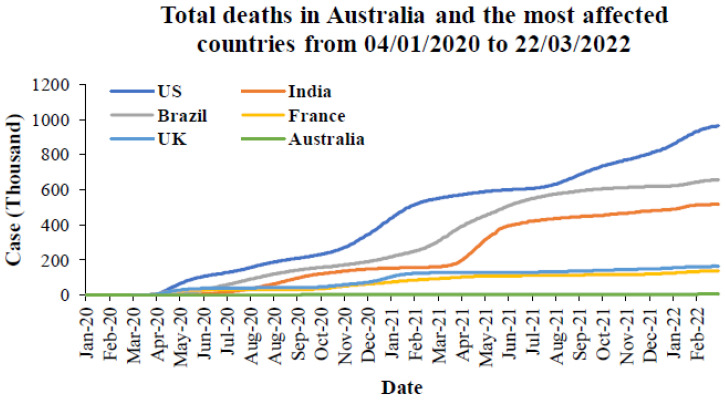
Total deaths in Australia and the most affected countries from 4 January 2020 to 22 March 2022 [64].

**Figure 15:**
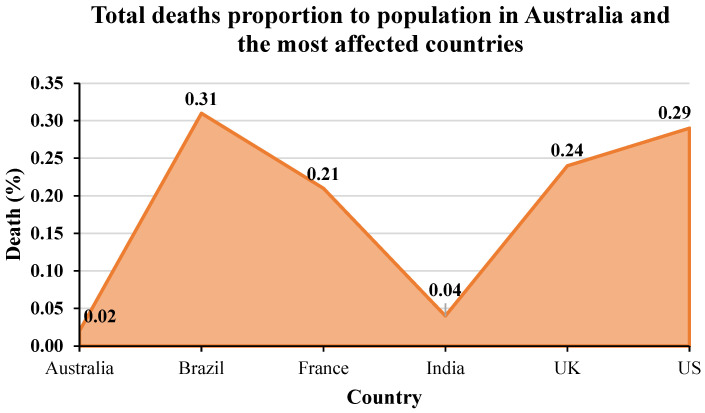
Total deaths proportion to population in Australia and the most affected countries from the first emergence to March 2022.

To understand the mortality caused by the COVID-19 pandemic, the total deaths in Australia and the most affected countries from the first emergence to March 2022 are provided in Figure 14. The meta-data from this figure were taken from the WHO [64]. From this figure, it can be seen that the US has the highest number of deaths followed by Brazil, India, the UK, and France. When comparing the total cases in Figure 3, India has higher cumulative cases than Brazil, followed by France. Australia has fewer deaths compared to the five most affected countries. However, when considering the proportion of the population in Figure 15, Brazil has the highest number of deaths, followed by the US, UK, and France. India and Australia have similar deaths in terms of the proportion of populations.

**Figure 16:**
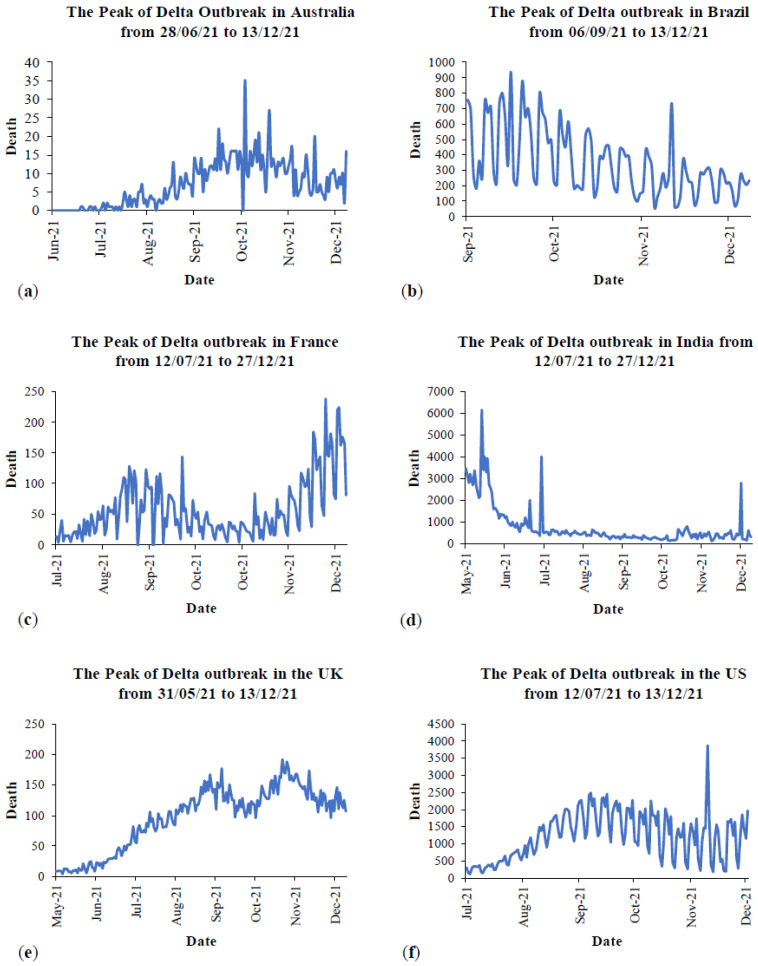
Mortality during the peak of Delta outbreaks in: (**a**) Australia, (**b**) Brazil, (**c**) France, (**d**) India, (**e**) the UK, and (**f**) the US [64].

**Figure 17:**
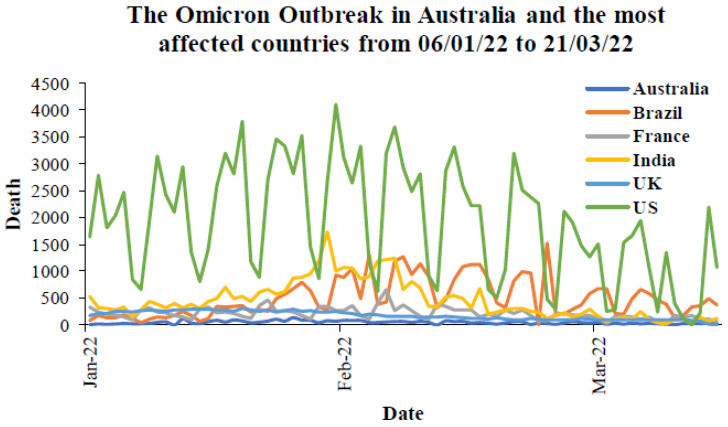
Mortality during the Omicron outbreaks in Australia and the most affected countries from 6 January to 21 March 2022 [64].

For more analysis, the daily deaths due to COVID-19 during the peak of the Delta outbreak in each country are provided in Figure 16. The peak period is taken from 80% to 100% of cases in total from Figure 11. Figure 17 presents the daily deaths during the Omicron outbreak from January to March 2022. For Australia (Figure 16a), the maximum number of daily deaths during the peak of the Delta outbreak was less than 40 cases, while there were over 100 deaths during the Omicron outbreak in Australia (Figure 17). For other countries, the maximum number of daily deaths during the peak of the Delta outbreak in Brazil (Figure 16b), France (Figure 16c), India (Figure 16d), the UK (Figure 16e), and the US (Figure 16f) were 935, 238, 6148, 192, and 3871 cases, respectively. During the Omicron outbreak in each country (Figure 17), the maximum number of daily deaths in Brazil, France, India, the UK, and the US were 482, 623, 2680, 280, and 4107 cases, respectively. It can be seen that Brazil and India had lower daily deaths during the Omicron outbreak, whereas other countries had higher daily deaths. However, the different mortalities might be because of the vaccination rate, other preventions for each country, and other parameters. The next section provides information on the prevention and the discussion of vaccination effectiveness.

## 3. Preventions

Currently, the SARS-CoV-2 epidemics are not over, and the Delta and Omicron variants have been circulating around the world with catastrophic consequences to societies and economies. In this consideration, it is extremely vital to continuously implement and practice safety prevention measures. For instance, there are several measures listed and discussed in Table 3, including vaccination effectiveness [86,87,88,89,90].

### Vaccination Programs

**Figure 18:**
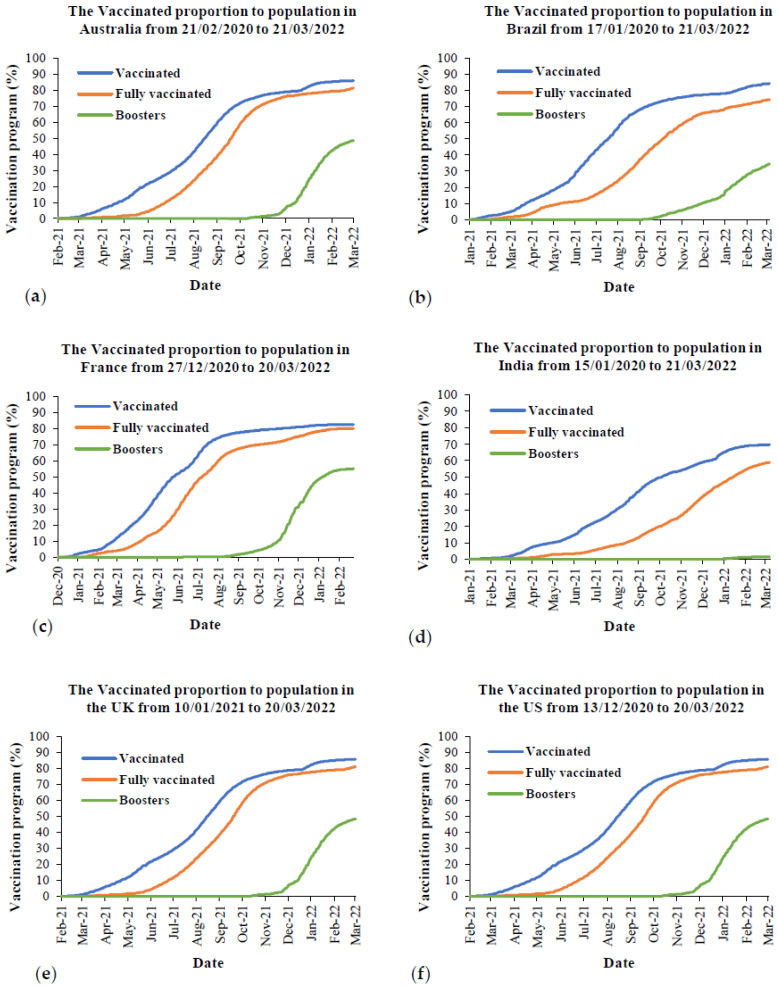
The vaccination programs in Australia and the most affected countries from the beginning to March 2022: (**a**) Australia, (**b**) Brazil, (**c**) France, (**d**) India, (**e**) the UK, and (**f**) the US.

One of the most effective ways to prevent people from SARS-CoV-2 is to become vaccinated. Figure 18 provides the vaccination programs from the first vaccination day to the current day for Australia and the five most affected countries. The meta-data from this figure were taken from Our World in Data [67]. From this figure, it is obvious that over 80% of the population of all countries is vaccinated, except for India, which is around 70% of the population. Regarding the fully vaccinated population, it is around 80% for the populations of Australia, France, the UK and the US, while it is around 74% and 59% for the populations of Brazil and India, respectively. For the boosters, it is around 50% for the populations of Australia, France, the UK and the US, and it is around 34% and 1% for the populations of Brazil and India, respectively.

To have insight into Delta variant transmissibility, it is necessary to analyse Massachusetts’s cluster, where there have been 469 cases since July 2021 during multiple summer events with huge public crowds. Notably, Massachusetts has a 69% fully vaccinated rate among its residents; however, in 469 cases, 346 cases occurred in fully vaccinated people, standing at 74% of total cases [92]. Thus, the Delta variant causes a great number of breakthrough infections, indicating its lofty contagion. When it comes to the severity and mortality profile of the latest Omicron variant, according to UK Health Security Agency [93], the risk of hospital admission from the Omicron variant is around one-third of the Delta variant. Moreover, they also suggest that the considerable reduction in hospitalization is attributed to three doses of the vaccine. However, it is highlighted that the dramatic number of infected cases is highly dedicated to a large number of hospital admissions, which steadily increases in the number of deaths. Thus, the Omicron variant is an actual threat to unvaccinated people.

The Delta variant, with a higher probability of hospitalization and ICU, leads an undoubted threat to unvaccinated people in the way that it spreads in low-vaccination areas. It can be explained by the fact that Australia had a low vaccination rate when the Delta variant hit the country, which was 24% including 5.8% and 18% for fully vaccinated and partially vaccinated people, respectively. When comparing the severity based on the peak of the Delta outbreak from Figure 13 to the vaccination programs, it was around 67% to 77% of the fully vaccinated population for all countries, except for India where it was around 41% of the fully vaccinated population. Thus, one of the possible factors causing a higher severity in both hospitalization and ICU due to the Delta variant is having a lower fully vaccinated rate. During the Omicron outbreak in each country, there was around 80% of the fully vaccinated population from all countries, except for India at around 50% fully vaccinated. Although there was a higher vaccination rate during the Omicron outbreak in all countries, the overall daily hospitalization during this period was still higher than the overall daily cases during the peak outbreak of the Delta variant. For the daily ICU cases, the overall daily ICU cases during the peak outbreak of the Delta variant was higher than the overall daily ICU cases during the Omicron outbreak for all countries (Figure 13). This was probably because of the higher vaccination rate. In terms of the mortality caused by the Delta and Omicron variants, there are limited indications that the Delta variant causes more deaths than other variants when it comes to mortality. However, it is accurate that the Delta variant has been currently posed to a high proportion of COVID-19 deaths among unvaccinated people [77,78]. When comparing the mortality from Figure 16 to the vaccination rate in Figure 18, the overall daily deaths during the Omicron outbreak, having a higher fully vaccinated rate, are still higher than the overall daily deaths during the peak of the Delta outbreak. This was observed in Australia, France, the UK, and the US. This might be because of the reduction of vaccine effectiveness due to the Omicron variant, prevention strategies, as well as the density of populations in each country.

## 4. Conclusions

In conclusion, the evidence to date demonstrates that severe acute respiratory syndrome coronavirus 2 (SARS-CoV-2) has been continuously evolving into new forms of variants. Unfortunately, these mutated variants with new infectious characteristics present a wide range of challenges in many countries. Although the Delta variant drastically increased in the number of infection waves in many countries in 2021, the Omicron variant has spread into over 63 countries since its initial discovery in November 2021 in South Africa. Conversely, by examining several recent infection waves in different countries with diverse backgrounds, there are some major characteristics of the Delta and Omicron variants listed below:

- The Delta variant is highly infectious, estimated to be more than double that of the previous variants.
- Fully vaccinated people can significantly become infected by the Delta variant versus other variants, known as breakthrough infections.
- The Omicron variant is currently the most divergent variant, with a high number of 26 unique mutations in the spike protein.
- There are limited data on the severity and mortality profile of the Omicron variant. However, based on the available meta-data from the validated resources, the Omicron variant significantly affected the increase in daily hospitalization compared to the daily cases during the peak of the Delta outbreak. However, the Omicron variant had less effect on the daily ICU case. A higher fully vaccinated rate is one of the main factors for the reduction of daily ICU cases recently.

However, the mortality rate during the Omicron outbreak is still higher than at the peak of the Delta outbreak. Even with a higher fully vaccinated rate, the mortality rate during this period is still higher than the previous Delta outbreak. This factor is still under investigation. The Delta variant can result in a great number of deaths, and there is still a shortage of confirmed information to indicate that the Delta variant causes a death toll in younger ages. Further reviews will consider more parameters that affect the mortality rate, especially at younger ages.

## Figures and Tables

**Table 1:** Recently designed Variants of Concern by WHO [32].

WHO Label	Pango Lineages	GISAID Clade	Next Strain Clade	Additional Amino Acid Changes Monitored	Earliest Documented Samples	Date of Designation
Alpha	B.1.1.7	GRY	201 (V1)	+S:484K +S:452R	United Kingdom, September 2020	18 December 2020
Beta	B.1.351 B.1.351.2 B.1.351.3	GH/501Y.V2	20H (V2)	+S:L18F	South Africa, May 2020	18 December 2020
Gamma	P.1 P.1.1 P.1.2 P.1.4 P.1.6 P.1.7	GR/501Y.V3	20J (V3)	+S:681H	Brazil, November 2020	11 January 2021
Delta	B.1.617.2 AY.1 AY.2 AY.3 AY.3.1	G/478K.V1	21A	+S 417N	India, October 2020	VOI: 4 April 2021 VOC: 11 May 2021
Omicron	B.1.1.529	GR/484A	21K	+G339D +S371L +S373L	Botswana, 11 November 2021	VOC: 26 November 2021

**Table 2:** Summary of mutational profile of spike mutations in the SARS-CoV-2 variants of concern (Alpha, Beta, Gamma, Delta and Omicron).

Variants	Total Spike Mutations	Unique Spike Mutations
Alpha	11	(4): A570D, D1118H, S982A, T716I
Beta	10	(6): A701V, D215G, D80A, ΔL242, ΔA243
Gamma	12	(8): D138Y, K417T, L18F, P26S, R190S, T1027I, T20N, V1176F
Delta	10	(7): D960N, E156G, L452R, P681R, T19R, ΔF157, ΔR158
Omicron	37	(26): A67V, D796Y, E484A, G339D, G446S, G496S, L212I, L981F, N440K, N679K, N764K, N856K, ins214EPE, N969K, Q493R, Q498R, Q954H, S371L, S373P, S375F, S477N, T547K, T95I, Y505H, ΔV143, ΔN211

**Table 3:** Delta variant preventive measures.

Measure	Discussion
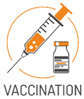 Getting Vaccinated	Current vaccines are partially remaining effective in protecting people from becoming infected or severely ill. According to analyses, the widely administered vaccines, Pfizer BioNTech and Oxford-AstraZeneca, have 96% and 92% effectiveness in preventing hospitalization after taking two doses, respectively [42,45,91]. Thus, the improvement of the vaccination rate is the most reliable solution to stop the current Delta variant pandemic.
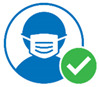 Wearing a mask	It is highly recommended to wear a well-fitted mask in all indoor places or areas of high or substantial transmission for unvaccinated people. For fully vaccinated people, it is essential to stop spreading the virus to other people by wearing a mask in all indoor places or areas of high or substantial transmission. Generally, there is no need to wear a mask in outdoor areas.
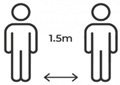 Practicing Social Distancing (1.5 m)	At residential areas, avoid close contact with infected family members; if possible, keep 1.5 m away from them. Outside residential areas, keep 1.5 m away with other people who you do not live with. Practicing social distancing is necessary to minimize the probability of becoming infected from people who are at risk.
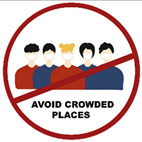 Avoiding overcrowded areas	In cases of easing restrictions involving opening restaurants, bars, fitness and gym centres, and theatres, being in these areas is supposed to be a higher risk for SARS-CoV-2. Especially, it is highly advised to avoid indoor areas with poor ventilation as much as possible.
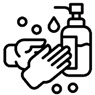 Thoroughly washing hands often	After contacting objects in public areas, thoroughly wash hands with soap as needed. In case of unavailable soap or water, wash hands with hand sanitizer made with at least 60% alcohol. Crucially, avoid touching your mouth, nose and eyes with unwashed hands
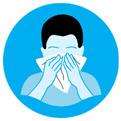 Covering sneezes and coughs	In cases of wearing a mask, people can sneeze or cough directly into their mask. Then, carefully clean their mask or put on a new one. In cases of not wearing a mask, use tissues or your elbow to cover mouth and nose when coughing or sneezing.
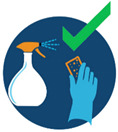 Regularly Cleaning and Disinfecting	In order to kill viruses and germs, regularly clean surfaces, such as tables, door handles, chairs, etc. Crucially, when there are infected people at home, it is highly recommended to disinfect all contacted surfaces listed above.
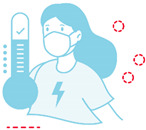 Monitoring health daily	Keep monitoring health daily, such as being aware of COVID-19 symptoms. In cases of developing symptoms, regularly check body temperature.

## Data Availability

Data will be available upon request.
